# Enthesitis in Psoriatic Arthritis, the Sonographic Perspective

**DOI:** 10.1007/s11926-021-01039-1

**Published:** 2021-08-24

**Authors:** Gianluca Smerilli, Andrea Di Matteo, Edoardo Cipolletta, Walter Grassi, Emilio Filippucci

**Affiliations:** 1 Rheumatology Unit, Department of Clinical and Molecular Sciences, Polytechnic University of Marche, “Carlo Urbani” Hospital, Via Aldo Moro 25, 60035 Jesi, Ancona, Italy; 2 Leeds Institute of Rheumatic and Musculoskeletal Medicine, University of Leeds, Leeds, UK

**Keywords:** Ultrasonography, Psoriatic arthritis, Seronegative spondyloarthritis, Enthesitis

## Abstract

**Purpose of Review:**

To provide an overview of the ultrasound (US) studies focusing on enthesitis in psoriatic arthritis (PsA).

**Recent Findings:**

Last-generation US equipment has demonstrated the ability to detect subtle morphostructural and vascular abnormalities at entheseal level. US is able to identify pathologic changes in both “classical” (i.e., the site of attachment of tendons, ligaments, and joint capsules into the bone) and “functional” entheses (i.e., anatomical regions where tendons or ligaments wrap around bony pulleys).

**Summary:**

US has the potential to be the first-line method in the assessment of enthesitis. In the present review we critically discussed the current definitions of US enthesitis, the scoring systems, and the main fields of application (i.e., the detection of enthesitis in PsA and psoriasis, the identification of different disease subsets, and the assessment of response to treatment).

## Introduction

The enthesis is the site of attachment of tendons, ligaments, and joint capsules into the bone [1]. It represents a fundamental link between the soft and force-generating tissues (i.e., muscles) and the hard scaffold of the body (i.e., bones).

Histologically, the entheses are classified as fibrous or fibrocartilaginous. The former are generally located at diaphyses or metaphyses of long bones (e.g., the deltoid insertion into the humerus), while the latter are characteristic of the tendons or ligaments that attach to epiphyses or apophyses (e.g., the Achilles tendon insertion into the calcaneal bone).

Clinically, fibrocartilaginous entheses represent the characteristic target of inflammation in patients with seronegative spondyloarthritis (SpA), including psoriatic arthritis (PsA) [2].

Furthermore, anatomical regions where tendons or ligaments wrap around bony pulleys are considered “functional entheses” albeit devoid of a direct attachment into bone, being sites of relevant mechanical stress leading to fibrocartilage differentiation [3]. Similarly to the fibrocartilaginous entheses, also functional entheses are targets of SpA [4].

Of note, the broad concept of “enthesis organ” highlights the importance of considering the enthesis not just as the focal anchoring site of tendons or ligaments. In fact, several tissues (fibrocartilage, trabecular bone, fat pat and synovial tissue of adjacent bursa/joint) contribute to mechanical stress dissipation [5]. The interplay between these components, in particular between synovial tissue of the adjacent bursa/joint and the enthesis itself (i.e., the “synovio-entheseal complex”), is a crucial element in the pathogenesis of SpA [6].

The term “enthesopathy” refers to any entheseal pathology, independently from the etiology which can be either traumatic, degenerative, inflammatory, or metabolic, while the term “enthesitis” entails the presence of inflammation at the enthesis, mainly in the context of seronegative SpA [1].

Enthesitis is a cardinal feature of PsA with a prevalence of approximately 30% when assessed by clinical examination [7–9]. It is part of the ClASsification for Psoriatic ARthritis (CASPAR) criteria [10], and it is one of the six accepted clinical domains to be considered when treating PsA patients according to the Group for Research and Assessment of Psoriasis and Psoriatic Arthritis (GRAPPA) [11]. In PsA, entheseal involvement has relevant therapeutic implications. According to the 2019 EULAR recommendations for the management of PsA, in patients with unequivocal enthesitis and insufficient response to nonsteroidal anti-inflammatory drugs (NSAIDs) or local glucocorticoid injections, therapy with a biologic disease-modifying antirheumatic drug (bDMARD) should be considered [12].

The clinical identification of enthesitis can be challenging [13, 14], and this has generated increasing interest in imaging assessment of this condition.

Conventional radiography (CR), while useful for the detection of long-standing entheseal pathology, has intrinsic limitations in the evaluation of enthesitis, because of poor depiction of soft tissue inflammation and low sensitivity in the identification of small entheseal bone erosions [15].

Magnetic resonance imaging (MRI) allows for a comprehensive assessment of enthesitis at both tendon and bony aspects, also at axial level; however, its main limitations include high costs, time required to examine multiple peripheral targets, and the need for high-end equipment [16]. Whole-body MRI might provide a fast assessment of several entheseal structures [17], but it is still far from a routine adoption in clinical practice.

Ultrasound (US) provides a detailed visualization of several morphostructural and vascular abnormalities indicative of entheseal active inflammation and structural damage [18–22]. It allows a real-time and feasible multi-site evaluation and has proven to be reliable in the assessment of peripheral entheses in SpA, including PsA [23–25]. The inability to detect bone marrow edema (i.e., osteitis) and the operator dependency represent the most important limitations of US in the assessment of enthesitis [26].

In view of the above, US has the potential to be the first-line method in the assessment of enthesitis [27].

The main aims of this review were to report a detailed overview of the milestone studies supporting the performances of US at entheseal level and to discuss critically its applications in daily rheumatology practice, with a focus on PsA.

## Ultrasound Definition of Enthesitis

Throughout the years, several US abnormalities have been described as part of the sonographic spectrum of enthesitis/enthesopathy in SpA. These include decreased echogenicity of the enthesis, entheseal thickening, enthesophytes, calcifications, bone erosions, cortical bone irregularities, perientheseal bursitis, and intra-tendinous, pre-insertional and intra-bursal power Doppler (PD) signal [28, 29].

**Fig. 1:**
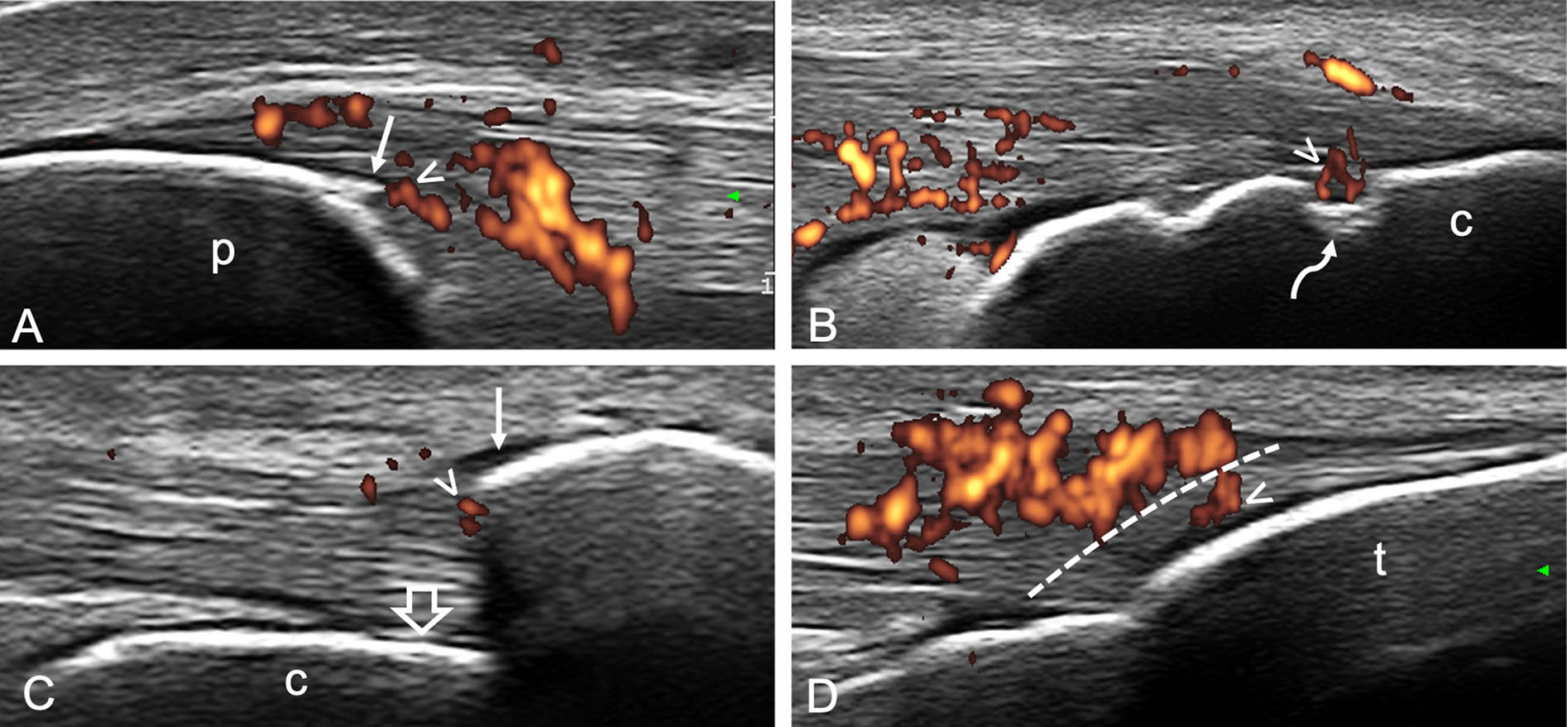
Longitudinal scans representative of enthesitis in psoriatic arthritis obtained with a 6–18-MHz probe at the proximal patellar tendon (**A**), Achilles tendon (**B**, **C**), and distal patellar tendon (**D**) insertions. Power Doppler signal (arrowhead) within 2 mm from the bony attachment is shown in **A**, **B**, **C**, and **D**. In **C**, power Doppler signal (score 1) is close to an exuberant enthesophyte (arrow) that disrupts the entheseal cortical line (open arrow) impairing its complete visualization. Of note, in **D**, only a small amount of PD signal (arrowhead) is in the region within 2 mm from the bony cortex (dashed line) even though a marked vascularization is present just proximal to it, leading to the same PD score shown in **C** (score 1). Note the proximity between power Doppler signal and signs of structural bone damage, such as enthesophytes (arrow) in **A** and **C** and a “hot” bone erosion (curved arrow) in **B**. c = calcaneus, p = patella, t = tibial tuberosity

In 2018, the following US definition of enthesitis in SpA/PsA was proposed by the Outcome Measures in Rheumatology (OMERACT) US Task Force: “hypoechoic and/or thickened insertion of the tendon close to the bone (within 2 mm from the bony cortex) which exhibits Doppler signal if active and which may show erosions and enthesophytes/calcifications as a sign of structural damage” [24] (Fig 1).

Such a definition, which was undoubtedly a step towards the standardization of entheseal US presents some issues which need to be further addressed.

First, the high prevalence of US pathologic findings at entheseal level in healthy subjects and in patients with metabolic syndrome undermines its specificity. In fact, at least one US abnormality was present in 73.4% of a cohort of 64 healthy subjects, being enthesophyte/calcification of quadriceps tendon and Achilles tendon insertions the most frequent findings [30]. Moreover, in a recent study of our group focusing on the five main lower limb entheses (i.e., the quadriceps insertion into the upper pole of the patella, the proximal and distal insertions of the patellar tendon, the calcaneal insertion of the Achilles tendon, and the plantar fascia), one or more US features of entheseal inflammation (i.e., entheseal thickening, hypoechogenicity, or PD signal) were found in at least one site in 30 out of 82 healthy subjects (34.1%) [31]. Noteworthy, the prevalence of PD signal was lower than those of entheseal thickening and hypoechogenicity, and PD grades > 1 were found in only one enthesis in a single healthy subject. In this study, our group proposed a new “cut-off” for the definition of “active” enthesitis, which should include a combination of gray-scale abnormalities and PD signal (i.e., PD signal ≥ 1 + entheseal thickening and/or hypoechogenicity), as well as considering as pathological only PD grades higher than 1 [32]. Similar results were also found in a study by Bakirci et al. assessing the same set of entheses plus the insertion of triceps tendon in 80 healthy subjects [33].

Finally, in a recent study, the presence of US findings indicative of enthesitis (i.e., entheseal thickening and hypoechoic areas at the entheseal level) were found in at least one enthesis in 38 (76%) out of 50 dysmetabolic patients [32, 34].

**Fig. 2:**
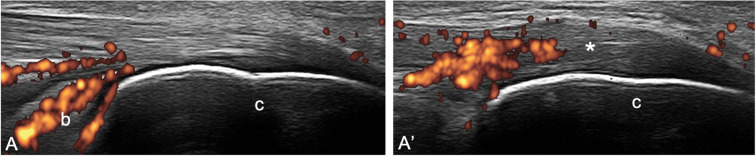
Right-left comparison of the Achilles tendon insertion in a patient with psoriatic arthritis presenting bilateral calcaneal tenderness. Longitudinal scans obtained with a 6–18-MHz probe. In both **A** and **A’**, power Doppler distribution does not fulfill the OMERACT definition “active” enthesitis (not being within 2 mm from the bony attachment). A deep retrocalcaneal bursitis (b) and a normal tendon are shown in **A**. In **A’** hypoechogenicity and thickening (*) can be appreciated compared to the contralateral side. c = calcaneus

**Fig. 3:**
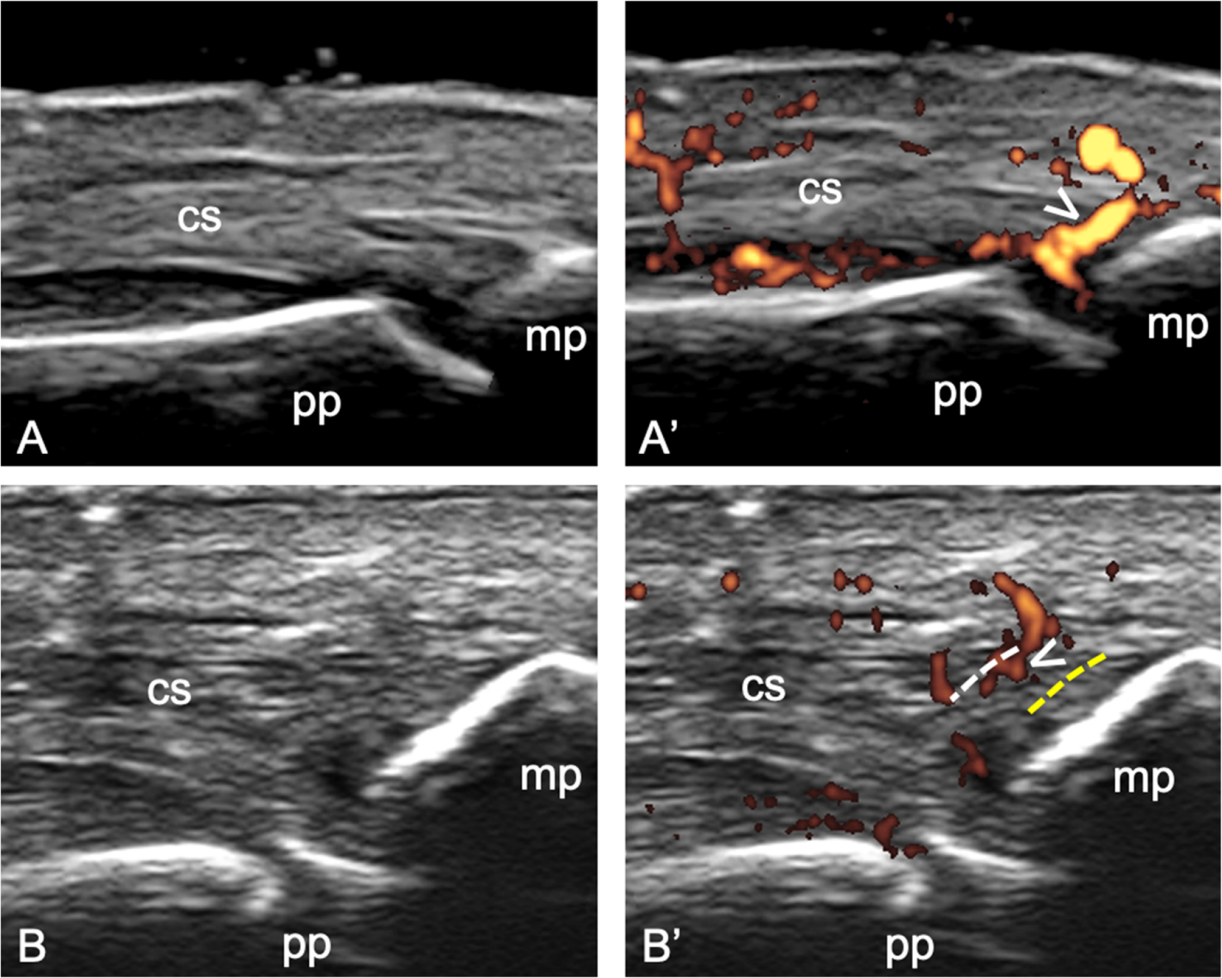
Longitudinal dorsal scans obtained with a 22-MHz probe at the proximal interphalangeal joint level in two psoriatic arthritis patients, without (**A** and **B**) and with (**A’** and **B’**) power Doppler mode, respectively. Distinct patterns of vascularization at the central slip (cs) of the finger extensor tendon insertion into the middle phalanx (mp) are shown. In **A’**, power Doppler signal (arrowhead) is close to the enthesis, while in **B’**, although being within 2 mm from the bony cortex (dashed line), the pre-insertional region is spared. We hypothesize that the optimal cut-off might be the half of the tendon thickness (yellow dashed line) in order to differentiate these two vascular patterns at small entheses level. pp = proximal phalanx

Second, the adoption of the 2-mm cut-off for the identification of entheseal PD signal to favor specificity may lead to the loss of precious information, since PD signal has frequently been detected outside the 2 mm area in patients with SpA [24, 25] (Fig 2). Thus, in the presence of PD signal close to the bone, also PD signal at tendon level may be considered an expression of enthesitis. Moreover, this cut-off has been developed by the OMERACT US Task Force on large entheses, mainly of the lower limb, and may not be applicable to the small entheses of the hands and feet. The entheses of the hands have been recently recognized as important targets in PsA [35–39]. An alternative option could be that the optimal cut-off varies according to the thickness of the tendon examined (e.g., PD signal not farther than half the entheseal thickness) (Fig 3). Furthermore, enthesophytes may disrupt the entheseal line impairing the clear visualization of the site where to place the caliper to measure the distance from the bony edge in order to delimit the area of interest where to detect Doppler signal. We believe that the area where to detect PD should move proximally together with the bony edge (i.e., the 2-mm distance should be measured from the tip of the enthesophyte) (Fig. 1C)

## Ultrasound Scoring Systems for Enthesitis

Over the last two decades, a number of scoring systems have been proposed for the quantification of entheseal burden of pathology at patient level in Spa and PsA and Table 1 reports a list of the most important indices.

**Table 1:** Main sonographic indices for US assessment of entheses in patients with seronegative spondyloarthritis (SpA) and psoriatic arthritis (PsA)

Author and index name	Year	Developed in patients with	Studied entheses	Scoring system	Time needed according to the developers	Discriminant validity
Balint et al. GUESS [19]	2002	SpA	QT, proximal and distal PT, AT, and PF	GUESS score (0 to 36), each item scores 1 point QT enthesis: tendon thickness > 6.1 mm, suprapatellar bursitis, bone erosion, enthesophyte Proximal PT enthesis: tendon thickness > 4 mm, bone erosion, enthesophyte Distal PT enthesis: tendon thickness > 4 mm, infrapatellar bursitis, bone erosion, enthesophyte AT enthesis: tendon thickness > 5.29 mm, retrocalcaneal bursitis, bone erosion, enthesophyte PF enthesis: PF thickness > 4.4 mm, bone erosion, enthesophyte	15 min	N.A.
D’Agostino et al. [21]	2003	SpA	CET, CFT, pubis, greater trochanter, QT, proximal PT, A, PF, TAT	Stage 1: Vascularization at the cortical junction without abnormal findings in gray scale Stage 2a: Vascularization associated with swelling and/or decreased echogenicity at the cortical junction in gray scale Stage 3a: Same as stage 2a, plus erosions of cortical bone and/or calcification of enthesis, and optional surrounding bursitis Stage 2b: Abnormal findings in B mode as in stage 2a, but without vascularization Stage 3b: Abnormal findings in B mode as in stage 3a, but without vascularization	20 min	N.A.
De Miguel et al. MASEI [40]	2009	SpA	TT, QT, proximal and distal PT, AT, and PF	MASEI score (0 to 136) Calcifications, Doppler signal, and erosions are scored on a semiquantitative score of 0 to 3 Tendon structure, tendon thickness, and bursitis (deep infrapatellar and retrocalcaneal) are either 0 or 1. Tendon structure was defined as pathological if loss of fibrillar pattern, hypoechoic aspect, or fusiform thickening of the enthesis occurred. Of note, enthesophytes and ossifications were included as calcifications	20 min	*SpA vs HS.* MASEI 18 points: Se 83.3%, Sp 82.2%, LR+ 4.8 [40]. *PsA vs HS*. MASEI 20 points: Se 30%, Sp 95%, LR+ 5.8 [41].
Filippucci et al. [23]	2009	SpA	AT	Soft tissue inflammation (seven items): tendon hypoechogenicity, entheseal hypoechogenicity, bursal effusion, PD signal at tendon level, PD signal at entheseal level, PD signal at bursal level Tissue damage (five items): intratendinous calcifications, entheseal calcifications, enthesophytes, bone erosions, bone irregularities* (not used to calculate total score) Two scores were proposed: [1] S total score for soft tissue inflammation, which resulted from the sum of the scores assigned to the 7 US findings indicative of soft tissue inflammation, ranging from 0 to 7 with presence/absence data and from 0 to 14 with semiquantitative scores [2] A total score for tissue damage, which resulted from the sum of the scores assigned to the 4 US findings indicative of tissue damage, ranging from 0 to 4 with presence/absence data and from 0 to 8 with semiquantitative scores.	N.A.	N.A.
Milutinovic et al. BUSES [42]	2015	SpA	CET, QT, proximal and distal PT, AT, and PF	BUSES score (0 to 132). Entheseal thickening, hypoechogenicity combined with lack of the normal fibrillar pattern, and enthesophytes were scored as 0 or 1, while Doppler signal at enthesis (up to 2 mm from the bony cortex) and bone erosions were scored as 0 or 4 points	N.A.	*SpA vs patients with enthesitis symptoms without SpA.* BUSES 7 points: Se 47.4%, Sp 90.2%, LR+ 4.83
Tom et al. GRAPPA Preliminary Ultrasonographic Enthesitis Score [43]	2019	PsA	ST, CET, proximal and distal PT, AT, and PF	The sonographic elemental entheseal lesions assessed at each enthesis are: hypoechogenicity, thickening, enthesophytes, bone erosions, and Doppler signal Doppler signal and enthesophytes were scored either by severity on a scale of 0–3 or as present/absent, the remaining variables were scored as present/absent. Doppler signal was considered positive if present within 5 mm from of the cortical bone or within the adjacent bursa.	N.A.	*PsA vs HS*. AUC is 0.93-0.94. A cut-off to evaluate optimal Se, Sp and LR+ has not been defined yet.

**Abbreviations.** AT = Achilles tendon insertion, BUSES = Belgrade Ultrasound Enthesitis Score, CET = common extensor tendon insertion into elbow lateral epicondyle, CFT = common flexor tendon insertion into elbow medial epicondyle, GRAPPA = Group for Research and Assessment of Psoriasis and Psoriatic Arthritis, GUESS = Glasgow Ultrasound Enthesitis Scoring System, HS = healthy subjects, LR+ = positive likelihood ratio, MASEI = MAdrid Sonographic Enthesitis Index, PF = plantar fascia, PsA = psoriatic arthritis, PT = patellar tendon insertion, QT = quadriceps tendon insertion, Se = sensitivity, Sp = specificity, SpA = seronegative spondyloarthritis, ST = supraspinatus tendon insertion, TAT = tibialis anterior tendon insertion, TT = triceps tendon insertion

Most of the systems included several morphostructural and Doppler abnormalities and a different set of entheses to examine. The concept of entheseal scoring systems itself implies the need for an extensive number of entheses to be scanned, and this impairs their routine adoption in most clinical settings for reasons of time. One possible solution could be a clinically driven scanning protocol, but this would lead to the loss of relevant information (i.e., subclinical enthesitis). However, during follow-up, the number of entheses to scan may be reduced to those most inflamed at baseline.

The first US scoring system specifically developed for assessing enthesitis in PsA was recently proposed by the GRAPPA US group [43]. They adopted a mixed expert and data-driven approach to define the elementary US abnormalities and entheseal sites to be included in a preliminary proposed US index. The authors tested the performance of this scoring system in distinguishing between 50 PsA patients and 50 age- and sex-matched healthy controls. The area under the ROC curve for the model was 0.93 if all abnormalities were scored as present/absent and 0.94 if PD signal and enthesophytes were scored on a 0–3 scale. This was the first effort to develop a US enthesitis score which included several experts from different research centers. Its main drawback might be the inclusion of the supraspinatus tendon insertion into the humerus greater tuberosity, which is a common site of pathology even in patients without PsA [44].

US assessment of the entheses has been incorporated also in composite sonographic scores, including other domains of the psoriatic disease.

The “Five Targets PD for Psoriatic Disease” score is based on PD US of joints, tendons, entheses, skin, and nails. The target with the highest expression of PD signal, one for each target area, is selected to be scanned at baseline and at follow-up assessments, providing a feasible and reliable approach for multi-target monitoring of psoriatic disease [45, 46].

Two US composite scores were developed by Ficjan et al. assessing 22 bilateral joints/entheses (PsASon22) and 13 unilateral joints/entheses (PsASon13) in patients with PsA [47]. The included entheses were the common extensor tendon origin at the lateral epicondyle of the humerus and the distal insertion of the patellar tendon into the anterior tibial tuberosity.

## The Ultrasound Detection of Enthesitis in Psoriatic Arthritis

Clinical examination performances in the assessment of enthesitis are poor when compared with US [13]. Some of the clinical signs of inflammation such as swelling, redness, and heat are frequently lacking even in large and superficial entheses (e.g., the Achilles insertion into the calcaneal bone), being rarely helpful. The clinical detection of enthesitis basically relies on tenderness to palpation, which is included in the most popular clinical enthesitis indices [e.g., the Leeds Enthesitis Index (LEI) and the Spondyloarthritis Research Consortium of Canada (SPARCC) Enthesitis Index] [48, 49].

However, in the clinical setting of entheseal pain, to distinguish “true” enthesitis from central sensitization is often difficult. Of note, while a higher number of tender entheses may be found in fibromyalgia than in PsA (mean Maastricht Ankylosing Spondylitis Enthesitis Score of 4.2 vs 1.9) [50], US signs of entheseal involvement are more frequent in PsA when compared to fibromyalgia (at least one enthesis affected in 90% of PsA patients vs 75% of fibromyalgia patients) [51].

The detection of subclinical enthesitis in PsA patients represents another relevant US application on top of clinical examination. Michelsen et al. performed a cross-sectional evaluation to compare clinical and US examination of Achilles enthesis in 141 patients with PsA [52]. Their results showed a lack of association between any of the US elementary findings and clinical enthesitis. Interestingly, the prevalence of subclinical US-detected inflammatory involvement in Achilles entheses without clinical enthesitis was 16%.

Moreover, in the case of unequivocal enthesitis, US can provide further information on the top of clinical examination, allowing for a quantitative assessment of the entity of the inflammation and revealing structural damage at entheseal level.

## Sonographic Enthesitis and Psoriatic Arthritis Severity

PsA is a multi-faceted disease, characterized by a considerable variability in terms of inflammation and consequent damage, ranging from oligosymptomatic involvement to a destructive arthropathy, in which the various domains of the psoriatic disease may be differently combined. Evidence is growing on the possible link between entheseal and joint pathology, in particular US enthesopathy has been correlated with a higher burden of radiographic damage at both axial and peripheral level [53–57].

A cross-sectional analysis conducted by Polachek et al. in 223 PsA patients revealed a positive correlation between a higher value of MAdrid Sonographic Enthesitis Index (MASEI) and hands and feet radiographic joint damage assessed by the modified Steinbrocker score (mSS): a 10-unit increase in MASEI value was associated with a 42% higher mSS. A higher MASEI value was also positively correlated with arthritis mutilans and with spine radiographic damage assessed by the modified Stoke Ankylosing Spondylitis Spine Score (mSASSS) [53]. Furthermore, the same group highlighted that the presence of HLA-B27 was associated with a higher value of MASEI in a cohort of 225 PsA patients [58].

The relationship between axial and entheseal domains was confirmed by Ruyssen-Witrand et al., who found an association between mSASSS and entheseal pathology detected by US at proximal and distal patellar tendon insertions, Achilles tendon insertion, and lateral epicondyle of the humerus. Interestingly, when analyzing each different structural abnormality, the strongest association was the one between mSASSS and the presence of at least one enthesophyte. The prevalence of syndesmophytes was higher in patients with than in those without US evidence of enthesophytes (26% vs 6%) [54].

Furthermore, a very recent study by Lackner et al. reported that US baseline enthesophytes at the MASEI entheseal sites were predictive of radiographic progression at entheseal level after 12 months in a cohort of 43 PsA patients [55].

The phenotyping of PsA patients is a long-standing dilemma; however, these recent contributes are starting to delineate a potential role for entheseal US to identify PsA subsets with different disease severity and damage, at both axial and peripheral levels.

## Monitoring Response to Treatment

As previously mentioned, enthesitis is one of the domains to be considered when treating patients with PsA [11]. In the last few years, all randomized controlled trials testing the efficacy of disease-modifying antirheumatic drugs (DMARDs) in PsA have included one or more clinical enthesitis measures as secondary outcomes [59, 60]. However, clinical examination is not sensitive neither specific for the detection of active enthesitis [13, 14].

Even if the vast majority of the published articles have focused on diagnostic or prognostic capabilities, US has also proven to be sensitive to change in SpA patients starting a bDMARD [61, 62]. Aydin et al. demonstrated the sensitivity to change of US inflammatory findings at Achilles enthesis level in 43 ankylosing spondylitis patients 2 months after the start of an anti-TNF treatment [61]. Naredo et al. conducted a prospective study on 327 patients with SpA starting anti-TNF treatment and confirmed that US findings indicative of entheseal inflammation were sensitive to change after 6 months [62].

On the other hand, only few pilot studies have assessed the US ability to detect treatment induced changes at entheseal level in a limited number of PsA patients [63, 64].

Acquacalda et al. performed a gray-scale and PD US assessment of Achilles tendon insertion, plantar fascia insertion, quadriceps tendon insertion, proximal patellar tendon insertion, and brachial triceps tendon insertion at baseline and after 6 months in a mixed cohort composed by 22 psoriasis (PsO) and 12 PsA patients starting a DMARD for a dermatologic indication. The authors found a non-significant improvement of US entheseal pathology, even if this study was underpowered by the low numerosity (only 23 patients completed the follow-up) and by the fact that none of the patients exhibited entheseal PD signal at baseline [63].

Litinsky et al. compared the effect of methotrexate (19 patients) and adalimumab (24 patients) in PsA. The scanning protocol was quite unusual, assessing only tendon thickness and including entheses (Achilles tendon and plantar fascia calcaneal insertions), tendons without synovial sheath (extensor digitorum tendons at the level of 2nd and 3rd metacarpophalangeal joints) and tendons with synovial sheath (flexor digitorum tendons at the level of 2nd and 3rd metacarpophalangeal joints). They found a trend towards a higher reduction in thickness of Achilles tendon and plantar fascia in the adalimumab group, even if the lack of PD examination represents a relevant limitation of this study [64].

Literature data are lacking on the possible differences of efficacy of bDMARDs on enthesitis using US as a reference method, as well as on the asynchrony between clinical and US and between articular and entheseal responses in PsA. It would be crucial to fill this gap of knowledge in order to better understand this multi-faceted disease and to offer a “personalized” treatment to PsA patients.

## Ultrasound Assessment of Functional Entheses

**Fig. 4:**
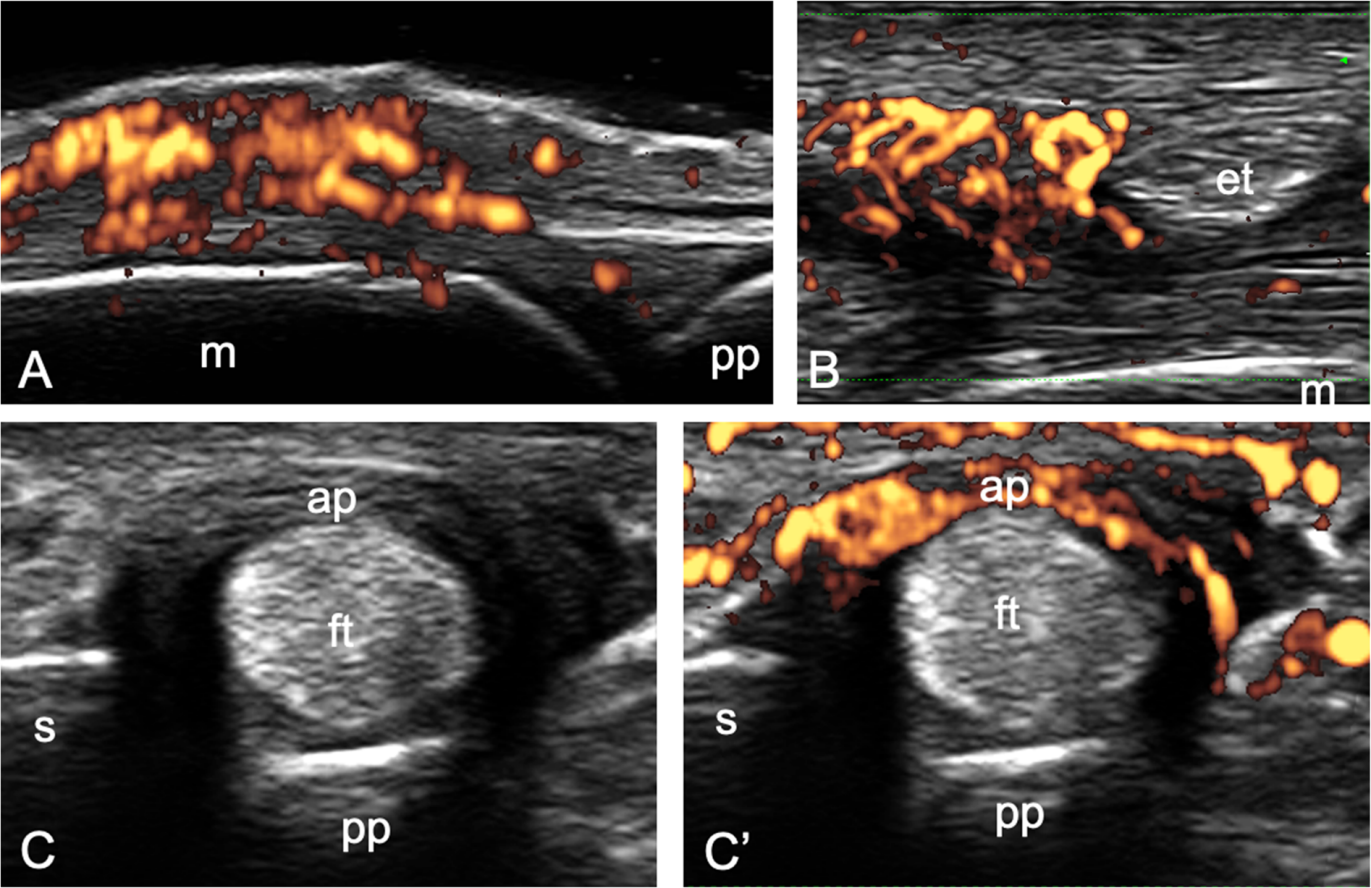
Functional enthesitis in psoriatic arthritis. In **A** and **B**, longitudinal and transverse scans obtained at the dorsal aspect of a metacarpophalangeal joint with 6–18-MHz and 22-MHz probes show a peritenon extensor tendon inflammation (PTI) pattern. In **C** and **C’**, transverse scans obtained at the volar aspect of a metacarpophalangeal joint of another patients with a 22-MHz probe show A1 pulley (ap) inflammation (power Doppler signal inside a thickened pulley). et = extensor tendon, ft = finger flexor tendons, m = metacarpal bone, pp = proximal phalanx, s = sesamoid bone

US has proven to be capable of identifying inflammatory changes not only at “classical entheses.” In fact, several functional entheses, especially at hand level, have been recognized as PsA targets and their morphostructural and vascular abnormalities can be reliably depicted by high-frequency US [36, 37, 65–68] (Fig. 4).

In 2011, Gutierrez et al. for the first time described an extraarticular inflammation detectable by US on the dorsal aspect of the metacarpophalangeal joint in PsA patients. It was named peritenon extensor tendon inflammation (PTI) and defined as “hypoechoic swelling of the soft tissue surrounding the extensor digitorum tendon, with or without peri-tendinous PD signal” [65]. This US finding was later confirmed in further studies and has been interpreted as a functional enthesitis in light of previous anatomical studies demonstrating the presence of fibrocartilage within the extensor tendon at metacarpophalangeal joint level [2, 36, 37, 66, 69]. Interestingly, the “entheseal” hypothesis about the site of this inflammation has been recently reinforced by the fact that a correlation between the presence of PTI and a higher MASEI was found in PsA patients by Macía-Villa and colleagues [70]. Of note, this sonographic pattern, which had traditionally been considered quite characteristic of psoriatic arthritis, has been recently described also in other diseases, such as systemic lupus erythematosus (SLE), rheumatoid arthritis, and palindromic rheumatism [71–73].

Annular pulleys, located on the volar aspect of the fingers in close relationship with finger flexor tendons, are functional entheses, being subjected to repetitive microtrauma and at least partially composed by fibrocartilaginous tissue [2, 74]. There is evidence of thickening of these structures in PsA patients [75], especially in those with previous history of dactylitis [68], compared with patients with PsO, rheumatoid arthritis, and healthy controls. Moreover, two very recent studies documented by US an inflammatory involvement of annular pulleys, defined as presence of PD signal within a thickened pulley, in PsA patients with and without dactylitis [39, 67].

Thus, a comprehensive US assessment of entheseal involvement in PsA patients should include functional entheses.

## Subclinical Enthesitis in Psoriasis

PsA has a prevalence of 6–42% among patients with PsO and skin involvement precedes joint disease in approximately 85% of the cases [76, 77]. There is evidence supporting the existence of a phase prior to the diagnosis of PsA characterized by the presence of nonspecific musculoskeletal symptoms (including heel pain) in PsO patients [78]. Therefore, an US assessment in PsO patients may provide a pictorial insight into the “psoriatic disease continuum”.

US has shown a higher prevalence of subclinical enthesitis in PsO patients compared to healthy controls and patients with other skin diseases [79–83] (Table 2).

**Table 2:** Main studies assessing entheseal pathology in psoriasis (PsO) patients without psoriatic arthritis (PsA) by ultrasound (US)

**Authors**	**Year**	**PsO patients (n)**	**Control group (n)**	**Control group characteristics**	**Studied entheses**	**PD mode**	**Probe frequency**
Gisondi et al. [79]	2008	30	30	Patients with dermatological diseases other than psoriasis	QT, proximal and distal PT, AT, and PF	No	10–15 MHz
Gutierrez et al. [80]	2011	45	45	Healthy controls	QT, proximal and distal PT, AT, and PF	Yes	6–18 MHz
Naredo et al. [81]	2011	136	46	Patients with dermatological diseases other than psoriasis	Deep finger flexor tendons, proximal and distal PT, AT, and PF	Yes	8–14 MHz
Zuliani et al. [82]	2019	40	20	Healthy controls	CET, QT, proximal and distal PT, AT, and PF	Yes	6–18 MHz

**Abbreviations.** AT = Achilles tendon insertion, CET = common extensor tendon insertion into the lateral epicondyle, PD = power Doppler, PF = plantar fascia insertion, PT = patellar tendon insertion, QT = quadriceps tendon insertion, TT = triceps tendon insertion

Gisondi et al. found a significantly higher Glasgow Ultrasound Enthesitis Scoring System (GUESS) score (7.9 vs 2.9) in 30 patients with PsO compared with 30 age- and sex-matched controls affected by other skin diseases [79]. Of note, this PsO cohort was followed longitudinally for an average period of 3.5 years and baseline thickness of the quadriceps tendon enthesis was found to be an independent predictor of the development of PsA [84].

Gutierrez et al. performed a cross-sectional study in 45 PsO patients and 45 age- and sex-matched healthy controls. The five lower limb entheses included in the GUESS were examined. The authors detected a higher GUESS score in PsO patients than in healthy controls as well as a higher prevalence of entheseal PD signal in PsO. However, PD signal was present in only 4 out of 450 entheses in PsO and in none of the healthy subjects [80].

The subclinical entheseal involvement in PsO was further investigated in a multicenter study conducted by Naredo et al. in 136 patients with plaque PsO and 46 age-matched controls with other skin diseases. The scanning protocol included the insertions of the following tendons: proximal patellar tendon, distal patellar tendon, Achilles tendon, plantar fascia, and deep flexor tendons of the fingers. Quadriceps tendon insertion was not assessed. The authors found a higher prevalence of enthesopathy, defined as abnormally hypoechoic and/or thickened tendon at its bony insertion, in PsO compared to controls (62.5% vs 39.1%). Entheseal PD signal was found in 10 (7.4%) PsO patients and in none of the controls (p=0.5) [81].

Recently, Zuliani et al. performed a PD US assessment in 40 PsO patients and 20 healthy controls at the five entheseal sites included in the GUESS plus the common extensor tendon insertion into the lateral epicondyle of the humerus. Active enthesitis, defined as the presence of PD signal within 2 mm from bony attachment and hypoechogenicity, was found only in PsO patients, with a prevalence of 20% at the patient level [82].

The impact of disease-modifying drugs on the subclinical entheseal involvement in PsO patients is an emerging and fascinating field of research. In 2019, Savage and colleagues demonstrated that ustekinumab reduced the US inflammatory burden at entheseal level in 23 PsO patients with at least one inflammatory entheseal change according to OMERACT definitions. The authors performed an extended scanning protocol including the entheses of both upper (i.e., the flexor and extensor pollicis longus, flexor digitorum profundus, extensor digitorum, common extensor and flexor tendons, distal brachial triceps tendon) and lower (i.e., quadriceps tendon, patellar tendon proximal and distal insertions, Achilles tendon, plantar fascia and peroneus brevis tendon) limbs. The percentage of entheses with at least one inflammatory finding decreased from 24.2 to 14.0% by week 24 and to 10.4% by week 52 [85].

Even if data are still scarce, these results might be the first step towards a new strategy of PsO stratification according to subclinical involvement which may eventually lead to the identification of patients “at increased risk” for the future development of PsA [86, 87]. This may have important implications for designing clinical trials on disease prevention.

## Conclusions

In this review, we described the most relevant applications of US in the assessment of enthesitis in PsA, from early diagnosis of enthesitis to assessment of disease severity and treatment response, highlighting the potential predictive value of sub-clinical entheseal inflammation for the development of PsA in patients with PsO.

Several scoring systems and definitions for enthesitis have been proposed by important international US societies, such as GRAPPA and OMERACT. Further research is needed to clarify their impact on diagnosis (including differential diagnosis), prognosis, and therapy monitoring in patients with PsA.

## References

[1] McGonagle D, Benjamin M. Enthesopathies. In: Hochberg MC, Gravallese EM, Silman AJ, Smolen JS, Weinblatt ME, Weisman MH. Rheumatology, 7th edition. 7th ed. Elsevier, editor. 2018. pp 1082-9

[2] Benjamin M, McGonagle D (2001). The anatomical basis for disease localisation in seronegative spondyloarthropathy at entheses and related sites. J Anat.

[3] Benjamin M, Ralphs JR (1998). Fibrocartilage in tendons and ligaments - an adaptation to compressive load. J Anat.

[4] Benjamin M, McGonagle D (2009). The enthesis organ concept and its relevance to the spondyloarthropathies. Adv Exp Med Biol.

[5] Benjamin M, Moriggl B, Brenner E, Emery P, McGonagle D, Redman S (2004). The “enthesis organ” concept: why enthesopathies may not present as focal insertional disorders. Arthritis Rheum.

[6] Watad A, Cuthbert RJ, Amital H, McGonagle D (2018). Enthesitis: much more than focal insertion point inflammation. Curr Rheumatol Rep.

[7] Gladman DD, Chandran V (2011). Observational cohort studies: lessons learnt from the University of Toronto Psoriatic arthritis program. Rheumatology (Oxford).

[8] Polachek A, Li S, Chandran V, Gladman DD (2017). Clinical enthesitis in a prospective longitudinal psoriatic arthritis cohort: incidence, prevalence, characteristics, and outcome. Arthritis Care Res.

[9] Pittam B, Gupta S, Harrison NL, Robertson S, Hughes DM, Zhao SS (2020). Prevalence of extra-articular manifestations in psoriatic arthritis: a systematic review and meta-analysis. Rheumatology (Oxford).

[10] Taylor W, Gladman D, Helliwell P, Marchesoni A, Mease P, Mielants H (2006). Classification criteria for psoriatic arthritis: development of new criteria from a large international study. Arthritis Rheum.

[11] Coates LC, Kavanaugh A, Mease PJ, Soriano ER, Laura Acosta-Felquer M, Armstrong AW, Bautista-Molano W, Boehncke WH, Campbell W, Cauli A, Espinoza LR, FitzGerald O, Gladman DD, Gottlieb A, Helliwell PS, Husni ME, Love TJ, Lubrano E, McHugh N, Nash P, Ogdie A, Orbai AM, Parkinson A, O'Sullivan D, Rosen CF, Schwartzman S, Siegel EL, Toloza S, Tuong W, Ritchlin CT (2016). Group for Research and Assessment of Psoriasis and Psoriatic Arthritis 2015 Treatment Recommendations for Psoriatic Arthritis. Arthritis Rheum.

[12] Gossec L, Baraliakos X, Kerschbaumer A, De Wit M, McInnes I, Dougados M (2020). EULAR recommendations for the management of psoriatic arthritis with pharmacological therapies: 2019 update. Ann Rheum Dis.

[13] Helliwell PS (2019). Assessment of enthesitis in psoriatic arthritis. J Rheumatol.

[14] Mease P (2020). Enthesitis in psoriatic arthritis (Part 3): clinical assessment and management. Rheumatology (Oxford).

[15] Aguila Maldonado R, Ruta S, Valuntas ML, García M (2017). Ultrasonography assessment of heel entheses in patients with spondyloarthritis: a comparative study with magnetic resonance imaging and conventional radiography. Clin Rheumatol.

[16] Kaeley GS (2020). Enthesitis in psoriatic arthritis (Part 2): imaging. Rheumatology (Oxford).

[17] Lecouvet FE (2016). Whole-body MR imaging: musculoskeletal applications. Radiology..

[18] Terslev L, Naredo E, Iagnocco A, Balint PV, Wakefield RJ, Aegerter P, Aydin SZ, Bachta A, Hammer HB, Bruyn GAW, Filippucci E, Gandjbakhch F, Mandl P, Pineda C, Schmidt WA, D'Agostino MA, on behalf of the Outcome Measures in Rheumatology Ultrasound Task Force (2014). Defining enthesitis in spondyloarthritis by ultrasound: Results of a delphi process and of a reliability reading exercise. Arthritis Care Res.

[19] Balint PV, Kane D, Wilson H, McInnes IB, Sturrock RD (2002). Ultrasonography of entheseal insertions in the lower limb in spondyloarthropathy. Ann Rheum Dis.

[20] Delle Sedie A, Riente L, Filippucci E, Scirè CA, Iagnocco A, Gutierrez M, Valesini G, Montecucco C, Grassi W, Bombardieri S (2010). Ultrasound imaging for the rheumatologist XXVI. Sonographic assessment of the knee in patients with psoriatic arthritis. Clin Exp Rheumatol.

[21] D’Agostino MA, Said-Nahal R, Hacquard-Bouder C, Brasseur JL, Dougados M, Breban M (2003). Assessment of peripheral enthesitis in the spondylarthropathies by ultrasonography combined with power Doppler: a cross-sectional study. Arthritis Rheum.

[22] Kaeley GS (2020). Visualization of enthesitis by ultrasound: a key diagnostic tool in spondyloarthropathy diagnosis and management. Curr Rheumatol Rep.

[23] Filippucci E, Aydin SZ, Karadag O, Salaffi F, Gutierrez M, Direskeneli H, Grassi W (2009). Reliability of high-resolution ultrasonography in the assessment of Achilles tendon enthesopathy in seronegative spondyloarthropathies. Ann Rheum Dis.

[24] Balint PV, Terslev L, Aegerter P, Bruyn GAW, Chary-Valckenaere I, Gandjbakhch F, Iagnocco A, Jousse-Joulin S, Möller I, Naredo E, Schmidt WA, Wakefield RJ, D'Agostino MA, OMERACT Ultrasound Task Force members (2018). Reliability of a consensus-based ultrasound definition and scoring for enthesitis in spondyloarthritis and psoriatic arthritis: an OMERACT US initiative. Ann Rheum Dis.

[25] Macía-Villa C, Falcao S, Medina J, De Miguel E (2019). Ultrasonography of enthesis in psoriatic arthritis: a descriptive and reliability analysis of elemental lesions and power Doppler subtypes. Scand J Rheumatol.

[26] Eder L, Aydin SZ, Kaeley GS, Maksymowych WP, Østergaard M (2018). Options for assessing joints and entheses in psoriatic arthritis by ultrasonography and magnetic resonance imaging: How to Move Forward. J Rheumatol Suppl.

[27] Kaeley GS, Eder L, Aydin SZ, Gutierrez M, Bakewell C (2018). Enthesitis: a hallmark of psoriatic arthritis. Semin Arthritis Rheum.

[28] Wakefield RJ, Balint PV, Szkudlarek M, Filippucci E, Backhaus M, D’Agostino MA (2005). Musculoskeletal ultrasound including definitions for ultrasonographic pathology. J Rheumatol.

[29] Zabotti A, Bandinelli F, Batticciotto A, Scirè CA, Iagnocco A, Sakellariou G (2017). Musculoskeletal ultrasonography for psoriatic arthritis and psoriasis patients: a systematic literature review. Rheumatology (Oxford).

[30] Guldberg-Møller J, Terslev L, Nielsen SM, Kønig MJ, Torp-Pedersen ST, Torp-Pedersen A, Christensen R, Bliddal H, Ellegaard K (2019). Ultrasound pathology of the entheses in an age and gender stratified sample of healthy adult subjects: a prospective cross-sectional frequency study. Clin Exp Rheumatol.

[31] Di Matteo A, Filippucci E, Cipolletta E, Martire V, Jesus D, Musca A (2020). How normal is the enthesis by ultrasound in healthy subjects?. Clin Exp Rheumatol.

[32] Di Matteo A, Filippucci E, Cipolletta E, Grassi W. Reply to: high prevalence of ultrasound-defined enthesitis in patients with metabolic syndrome. Clin Exp Rheumatol. 2020 23. Online ahead of print. 32324124

[33] Bakirci S, Solmaz D, Stephenson W, Eder L, Roth J, Aydin SZ (2020). Entheseal changes in response to age, body mass index and physical activity: an ultrasound study in healthy people. J Rheumatol.

[34] Falsetti P, Conticini E, Baldi C, Acciai C, Frediani B. High prevalence of ultrasound-defined enthesitis in patients with metabolic syndrome. Comment on: How normal is the enthesis by ultrasound in healthy subjects? Di Matteo et al. Clin Exp Rheumatol. 2020. Online ahead of print. 32324127

[35] Filippou G, Di Sabatino V, Adinolfi A, Bertoldi I, Picerno V, Biasi G (2013). No enthesis should be overlooked when psoriatic arthritis is suspected: enthesitis of the extensor digitorum tendons. J Rheumatol.

[36] Zabotti A, Idolazzi L, Batticciotto A, De Lucia O, Scirè CA, Tinazzi I (2017). Enthesitis of the hands in psoriatic arthritis: an ultrasonographic perspective. Med Ultrason.

[37] Zabotti A, Salvin S, Quartuccio L, De Vita S (2016). Differentiation between early rheumatoid and early psoriatic arthritis by the ultrasonographic study of the synovio-entheseal complex of the small joints of the hands. Clin Exp Rheumatol.

[38] Tinazzi I, McGonagle D, Zabotti A, Chessa D, Marchetta A, Macchioni P (2018). Comprehensive evaluation of finger flexor tendon entheseal soft tissue and bone changes by ultrasound can differentiate psoriatic arthritis and rheumatoid arthritis. Clin Exp Rheumatol.

[39] Smerilli G, Cipolletta E, Di Carlo M, Di Matteo A, Grassi W, Filippucci E (2020). Power Doppler Ultrasound Assessment of A1 Pulley. A new target of inflammation in psoriatic arthritis?. Front Med (Lausanne).

[40] De Miguel E, Cobo T, Muñoz-Femández S, Naredo E, Usón J, Acebes JC (2009). Validity of enthesis ultrasound assessment in spondyloarthropathy. Ann Rheum Dis.

[41] Eder L, Jayakar J, Thavaneswaran A, Haddad A, Chandran V, Salonen D, Rosen CF, Gladman DD (2014). Is the MAdrid Sonographic Enthesitis Index useful for differentiating psoriatic arthritis from psoriasis alone and healthy controls?. J Rheumatol.

[42] Milutinovic S, Radunovic G, Veljkovic K, Zlatanovic M, Zlatkovic Svenda M, Perovic Radak M, Pavlov Dolijanovic S, Stojic B, Damjanov N (2015). Development of ultrasound enthesitis score to identify patients with enthesitis having spondyloarthritis: prospective, double-blinded, controlled study. Clin Exp Rheumatol.

[43] Tom S, Zhong Y, Cook R, Aydin SZ, Kaeley G, Eder L (2019). Development of a preliminary ultrasonographic enthesitis score in psoriatic arthritis - GRAPPA ultrasound working group. J Rheumatol.

[44] Iagnocco A, Filippucci E, Meenagh G, Delle Sedie A, Riente L, Bombardieri S, Grassi W, Valesini G (2006). Ultrasound imaging for the rheumatologist. I. Ultrasonography of the shoulder. Clin Exp Rheumatol.

[45] Gutierrez M, Filippucci E, De Angelis R, Filosa G, Kane D, Grassi W (2010). A sonographic spectrum of psoriatic arthritis: “the five targets”. Clin Rheumatol.

[46] Gutierrez M, Di Geso L, Salaffi F, Bertolazzi C, Tardella M, Filosa G (2012). Development of a preliminary US power Doppler composite score for monitoring treatment in PsA. Rheumatology (Oxford).

[47] Ficjan A, Husic R, Gretler J, Lackner A, Graninger WB, Gutierrez M, Duftner C, Hermann J, Dejaco C (2014). Ultrasound composite scores for the assessment of inflammatory and structural pathologies in Psoriatic Arthritis (PsASon-Score). Arthritis Res Ther.

[48] Healy PJ, Helliwell PS (2008). Measuring clinical enthesitis in psoriatic arthritis: assessment of existing measures and development of an instrument specific to psoriatic arthritis. Arthritis Rheum.

[49] Maksymowych WP, Mallon C, Morrow S, Shojania K, Olszynski WP, Wong RL, Sampalis J, Conner-Spady B (2009). Development and validation of the Spondyloarthritis Research Consortium of Canada (SPARCC) Enthesitis Index. Ann Rheum Dis.

[50] Marchesoni A, Atzeni F, Spadaro A, Lubrano E, Provenzano G, Cauli A (2012). Identification of the clinical features distinguishing psoriatic arthritis and fibromyalgia. J Rheumatol.

[51] Macchioni P, Salvarani C, Possemato N, Gutierrez M, Grassi W, Gasparini S, Perricone C, Perrotta FM, Grembiale RD, Bruno C, Tripolino C, Govoni M, Ciancio G, Farina I, Ramonda R, Frallonardo P, Desiati F, Scarpa R, Costa L, Zabotti A, de Vita S, D’Attino RM, Gualberti G, Merolla R, di Luzio Paparatti U, Aldigeri R, Marchesoni A (2019). Ultrasonographic and clinical assessment of peripheral enthesitis in patients with psoriatic arthritis, psoriasis, and fibromyalgia syndrome: the ULISSE study. J Rheumatol.

[52] Michelsen B, Diamantopoulos AP, Soldal DM, Hammer HB, Kavanaugh A, Haugeberg G (2017). Achilles enthesitis defined by ultrasound is not associated with clinical enthesitis in patients with psoriatic arthritis. RMD Open.

[53] Polachek A, Cook R, Chandran V, Gladman DD, Eder L (2017). The association between sonographic enthesitis and radiographic damage in psoriatic arthritis. Arthritis Res Ther.

[54] Ruyssen-Witrand A, Jamard B, Cantagrel A, Nigon D, Loeuille D, Degboe Y, Constantin A (2017). Relationships between ultrasound enthesitis, disease activity and axial radiographic structural changes in patients with early spondyloarthritis: data from DESIR cohort. RMD Open.

[55] Lackner A, Heber D, Bosch P, Adelsmayr G, Duftner C, Ficjan A (2020). Ultrasound verified enthesophytes are associated with radiographic progression at entheses in psoriatic arthritis. Rheumatology (Oxford).

[56] Ayan G, Tinazzi I, Bakirci S, Gazel U, Solmaz D, Kalyoncu U, et al. Disease activity at the entheses and joints are correlated in psoriatic arthritis when explored with ultrasound. Clin Exp Rheumatol. 2021. Online ahead of print. 10.55563/clinexprheumatol/frrq75 33427623

[57] Solmaz D, Bakirci S, Jibri Z, Sampaio M, Karsh J, Aydin SZ (2020). Psoriasis is an independent risk factor for entheseal damage in axial spondyloarthritis. Semin Arthritis Rheum.

[58] Polachek A, Cook R, Chandran V, Abji F, Gladman D, Eder L (2018). The Association Between HLA Genetic Susceptibility Markers and Sonographic Enthesitis in Psoriatic Arthritis. Arthritis Rheum.

[59] Mease P, Van Der Heijde D, Landewé R, Mpofu S, Rahman P, Tahir H (2018). Secukinumab improves active psoriatic arthritis symptoms and inhibits radiographic progression: primary results from the randomised, double-blind, phase III FUTURE 5 study. Ann Rheum Dis.

[60] Mease PJ, Gladman DD, Collier DH, Ritchlin CT, Helliwell PS, Liu L, Kricorian G, Chung JB (2019). Etanercept and methotrexate as monotherapy or in combination for psoriatic arthritis: primary results from a randomized, controlled phase III trial. A Arthritis Rheumatol.

[61] Aydin SZ, Karadag O, Filippucci E, Atagunduz P, Akdogan A, Kalyoncu U, Grassi W, Direskeneli H (2010). Monitoring Achilles enthesitis in ankylosing spondylitis during TNF-α antagonist therapy: an ultrasound study. Rheumatology (Oxford).

[62] Naredo E, Batlle-Gualda E, García-Vivar ML, García-Aparicio AM, Fernández-Sueiro JL, Fernández-Prada M, Giner E, Rodriguez-Gomez M, Pina MF, Medina-Luezas JA, Toyos FJ, Campos C, Gutiérrez-Polo R, Ferrer MA, Martínez O, Díaz-Torne C, Gonzalez T, Campos S, Queiro R, Castaño-Sánchez M, Aznar JJ, Bustabad S, Paez-Camino M, Tuneu R, Ruiz T, Mateo L, Pujol M, Ponce A, Ros I, Gallegos A, Moreno J, Gumbau D, Sianes M, Poveda-Elices MJ, Romero-Gómez M, Raya E, Ultrasound Group of the Spanish Society of Rheumatology (2010). Power Doppler ultrasonography assessment of entheses in spondyloarthropathies: response to therapy of entheseal abnormalities. J Rheumatol.

[63] Acquacalda E, Albert C, Montaudie H, Fontas E, Danre A, Roux CH, Breuil V, Lacour JP, Passeron T, Euller Ziegler L (2015). Ultrasound study of entheses in psoriasis patients with or without musculoskeletal symptoms: a prospective study. Joint Bone Spine.

[64] Litinsky I, Balbir-Gurman A, Wollman J, Arad U, Paran D, Caspi D, Elkayam O (2016). Ultrasound assessment of enthesis thickening in psoriatic arthritis patients treated with adalimumab compared to methotrexate. Clin Rheumatol.

[65] Gutierrez M, Filippucci E, Salaffi F, Di Geso L, Grassi W (2011). Differential diagnosis between rheumatoid arthritis and psoriatic arthritis: the value of ultrasound findings at metacarpophalangeal joints level. Ann Rheum Dis.

[66] Macía-Villa C, Falcao S, Gutierrez M, Medina J, Hammer HB, De Miguel E (2018). What is metacarpophalangeal joint swelling in psoriatic arthritis? Ultrasound findings and reliability assessment. Clin Exp Rheumatol.

[67] Tinazzi I, McGonagle D, Macchioni P, Aydin SZ (2020). Power Doppler enhancement of accessory pulleys confirming disease localization in psoriatic dactylitis. Rheumatology (Oxford).

[68] Tinazzi I, McGonagle D, Aydin SZ, Chessa D, Marchetta A, Macchioni P (2018). “Deep Koebner” phenomenon of the flexor tendon-associated accessory pulleys as a novel factor in tenosynovitis and dactylitis in psoriatic arthritis. Ann Rheum Dis.

[69] Milz S, Putz R, Ralphs JR, Benjamin M (1999). Fibrocartilage in the extensor tendons of the human metacarpophalangeal joints. Anat Rec.

[70] Macía-Villa C, Falcao S, Gutierrez M, Medina J, Hammer HB, de Miguel E (2019). Peritenon extensor tendon inflammation in psoriatic arthritis is an enthesitis-related lesion. J Rheumatol.

[71] Di Matteo A, De Angelis R, Cipolletta E, Filippucci E, Grassi W (2018). Systemic lupus erythematosus arthropathy: the sonographic perspective. Lupus..

[72] Mankia K, D’Agostino MA, Wakefield RJ, Nam JL, Mahmood W, Grainger AJ, Emery P (2019). Identification of a distinct imaging phenotype may improve the management of palindromic rheumatism. Ann Rheum Dis.

[73] Ogura T, Hirata A, Hayashi N, Takenaka S, Ito H, Mizushina K, Fujisawa Y, Imamura M, Yamashita N, Nakahashi S, Kujime R, Kameda H (2017). Comparison of ultrasonographic joint and tendon findings in hands between early, treatment-naïve patients with systemic lupus erythematosus and rheumatoid arthritis. Lupus..

[74] Sampson SP, Badalamente MA, Hurst LC, Seidman J (1991). Pathobiology of the human A1 pulley in trigger finger. J Hand Surg [Am].

[75] Furlan A, Stramare R (2018). The thickening of flexor tendons pulleys: a useful ultrasonographical sign in the diagnosis of psoriatic arthritis. J Ultrasound.

[76] Gladman D, Antoni C, Mease P, Clegg DO, Nash P (2005). Psoriatic arthritis: epidemiology, clinical features, course, and outcome. Ann Rheum Dis.

[77] Catanoso M, Pipitone N, Salvarani C (2012). Epidemiology of psoriatic arthritis. Reumatismo..

[78] Eder L, Polachek A, Rosen CF, Chandran V, Cook R, Gladman DD (2017). The development of psoriatic arthritis in patients with psoriasis is preceded by a period of nonspecific musculoskeletal symptoms: a prospective cohort study. Arthritis Rheum.

[79] Gisondi P, Tinazzi I, El-Dalati G, Gallo M, Biasi D, Barbara LM (2008). Lower limb enthesopathy in patients with psoriasis without clinical signs of arthropathy: a hospital-based case-control study. Ann Rheum Dis.

[80] Gutierrez M, Filippucci E, De Angelis R, Salaffi F, Filosa G, Ruta S (2011). Subclinical entheseal involvement in patients with psoriasis: an ultrasound study. Semin Arthritis Rheum.

[81] Naredo E, Möller I, de Miguel E, Batlle-Gualda E, Acebes C, Brito E (2011). High prevalence of ultrasonographic synovitis and enthesopathy in patients with psoriasis without psoriatic arthritis: a prospective case-control study. Rheumatology (Oxford).

[82] Zuliani F, Zabotti A, Errichetti E, Tinazzi I, Zanetti A, Carrara G, Quartuccio L, Sacco S, Giovannini I, Stinco G, de Vita S (2019). Ultrasonographic detection of subclinical enthesitis and synovitis: a possible stratification of psoriatic patients without clinical musculoskeletal involvement. Clin Exp Rheumatol.

[83] Eder L, Aydin SZ (2018). Imaging in psoriatic arthritis—insights about pathogenesis of the disease. Curr Rheumatol Rep.

[84] Tinazzi I, McGonagle D, Biasi D, Confente S, Caimmi C, Girolomoni G (2011). Preliminary evidence that subclinical enthesopathy may predict psoriatic arthritis in patients with psoriasis. J Rheumatol.

[85] Savage L, Goodfield M, Horton L, Watad A, Hensor E, Emery P, Wakefield R, Wittmann M, McGonagle D (2019). Regression of peripheral subclinical enthesopathy in therapy-naive patients treated with ustekinumab for moderate-to-severe chronic plaque psoriasis: a fifty-two–week, prospective, open-label feasibility study. Arthritis Rheum.

[86] Perez-Chada LM, Haberman RH, Chandran V, Rosen CF, Ritchlin C, Eder L, Mease P, Reddy S, Ogdie A, Merola JF, Scher JU (2021). Consensus terminology for preclinical phases of psoriatic arthritis for use in research studies: results from a Delphi consensus study. Nat Rev Rheumatol.

[87] Zabotti A, Tinazzi I, Aydin SZ, McGonagle D (2020). From psoriasis to psoriatic arthritis: insights from imaging on the transition to psoriatic arthritis and implications for arthritis prevention. Curr Rheumatol Rep.

